# Nuclease-Treated Stabilized Fermentation Product of *Cetobacterium somerae* Improves Growth, Non-specific Immunity, and Liver Health of Zebrafish (*Danio rerio*)

**DOI:** 10.3389/fnut.2022.918327

**Published:** 2022-07-06

**Authors:** Mingxu Xie, Qiang Hao, Rui Xia, Rolf Erik Olsen, Einar Ringø, Yalin Yang, Zhen Zhang, Chao Ran, Zhigang Zhou

**Affiliations:** ^1^Sino-Norway Joint Lab on Fish Gut Microbiota, Institute of Feed Research, Chinese Academy of Agricultural Sciences, Beijing, China; ^2^Norway-China Joint Lab on Fish Gastrointestinal Microbiota, Institute of Biology, Norwegian University of Science and Technology, Trondheim, Norway; ^3^Faculty of Biosciences, Fisheries, and Economics, Norwegian College of Fisheries Science, UiT The Arctic University of Norway, Tromsø, Norway; ^4^Key Laboratory for Feed Biotechnology of the Ministry of Agriculture and Rural Affairs, Institute of Feed Research, Chinese Academy of Agricultural Sciences, Beijing, China

**Keywords:** *Cetobacterium somerae*, nuclease, growth, non-specific immunity, liver health

## Abstract

High-fat diets (HFD) are harmful to fish health. Probiotics are commonly utilized to improve fish nutrition metabolism, immune response, and health. Nucleic acids of the probiotic bacterium can be hydrolyzed by nuclease to generate nucleotides. The present study aimed to evaluate the effects of stabilized fermentation product of nuclease-treated *Cetobacterium somerae* XMX-1 [XMX-1 (N)] on growth, non-specific immunity, and liver health of zebrafish (*Danio rerio*). Compared to the HFD group, 100 g/kg XMX-1 (N) significantly increased weight gain and decreased feed conversion ratio (FCR). However, 5 or 10 g/kg XMX-1 (N) had no influence on zebrafish growth. In addition, supplementation of 100 g/kg XMX-1 (N) significantly increased lysozyme activity and total antioxidant capacity in skin mucus, and the expression of inflammation related genes interleukin 1 beta (*IL-1*β), interleukin 10 (*IL-10*), and interleukin 6 (*IL-6*) in the gut as well as fatty acid oxidation related genes uncoupling protein 2 (*UCP2*) and proliferator-activated receptor γ coactivator 1α (*PGC1*α) in the liver, while decreased the content of hepatic triacylglycerol (TAG) in zebrafish. The gene sequencing, 16S *r*RNA, showed that 100 g/kg XMX-1 (N) enhanced the relative abundance of Firmicutes while lowered Proteobacteria and Actinobacteria. 10 g/kg XMX-1 (N) significantly increased lysozyme activity and complement component 4 (C4) in skin mucus, and intestinal expression of inflammation-related genes. In the 5 g/kg XMX-1 (N) group, however, only an increase in C4 level in skin mucus was observed. Together, these results reveal that dietary supplementation with nuclease-treated *C. somerae* XMX-1 (N) has a dose-dependent beneficial effect on fish health.

## Introduction

With the widespread adoption of large-scale and intense fish culture practices, the phenomena of excessive body fat deposition in farmed fish have become quite frequent, resulting in fatty liver, which may affect fish health and reduce harvest yields (1–4). Excessive fat accumulation has been reported to cause hepatic and intestinal oxidative stress (5, 6), and affect normal gut microbiota leading to dysbiosis (7, 8). Therefore, it is significant to seek strategies to mitigate the negative consequences of high-fat diets. The previous studies have shown that probiotics can maintain gut microbiota homeostasis and ameliorate fatty liver, as well as improve liver function of fish (6, 9, 10). In addition, probiotics have been reported to improve growth, intestinal health, the immune system, and prevent diseases (11–14).

*Cetobacterium* is an indigenous dominant bacterium in the gut microbiota of fish (15, 16). *Cetobacterium* and its metabolite acetate improved glucose homeostasis through parasympathetic activation in zebrafish (17). In our previous studies, dietary supplemented fermentation product of *Cetobacterium somerae* can reverse the negative effect associated with higher plant proteins addition by improving gut and liver health, as well as reducing liver lipid deposition of common carp (*Cyprinus carpio*) (18). In zebrafish, the addition of stabilized fermentation product of *C. somerae* can improve gut and liver health and antiviral immunity, suggesting *C. somerae* can be used as a potential probiotic to improve fish health (19). Although the beneficial effects of *C. somerae* have been characterized, the associated effector molecules of *C. somerae* are largely unknown, and it is practical to improve the function of *C. somerae* fermentation product by a treatment targeting potential beneficial components.

Nucleic acids of the probiotic bacterium can be hydrolyzed by nuclease to produce nucleotides and other metabolites (20–23). Although nucleotides are low-molecular-weight compounds, they are extremely important in organisms and many biological activities rely on nucleotides and their associated metabolites (24, 25). Consistent with the results in mammals (26, 27), supplementation of nucleotides in fish and shrimp feed can stimulate growth, improve intestinal health, and boost anti-stress as well as immune responses (28). A recent study also showed that dietary nucleotides reduced hepatic steatosis in zebrafish (29).

The aim of the study is to further improve the availability of nucleotides in the fermentation product of *C. somerae* by nuclease treatment, and assess the effects of nuclease-treated *C. somerae* fermentation product on zebrafish health. To our knowledge, this is the first study about the beneficial role of the commensal bacteria *C. somerae* treated with nuclease in fish. Overall, the findings might help to promote the utilization of nuclease-treated *C. somerae* fermentation product as a feed supplement.

## Materials and Methods

### Bacteria Culture and Nuclease Treatment

*Cetobacterium somerae* XMX-1, with the preservation number CGMCC No. 18908 in the China General Microbiological Culture Collection Center, was anaerobically cultivated in an anaerobic incubator (Electrotek, England) growing in Glfu Anaerobic Medium (GAM) Broth medium (Haibo, China) at 28°C for 18 h to reach the concentration of 10^8^ CFU/ml. The protocols for obtaining stabilized fermentation product of *C. somerae* were conducted as previously described (19).

The stabilized fermentation product of *C. somerae* was treated with 3% nuclease (Nanning Pang Bo Bioengineering Co., Ltd., Guangxi, China) with the marker of 50,000 U/g in a 60°C incubator for 48 h. The concentration of nucleotides was measured at 48 h after treatment of ultrasonic crushing, which was shown as 58.2 μg/g. In contrast, the concentration of nucleotides was 44.2 μg/g without the treatment of nuclease. Proximate composition analysis of the fermentation product of *C. somerae* and nuclease-treated fermentation product of *C. somerae* was listed in Table 1. The moisture content was measured using the drying method (GB/T 6435-2014); crude ash was determined according to the ashing method (GB/T 6438-2007); crude fat was determined by the Soxhlet extraction method (GB/T 6433-2006); crude protein was measured by the Kjeldahl method (GB/T 6432-1994); crude fiber was measured using filter bag technology (GB/T 6434-2006). Finally, stabilized fermentation product of *C. somerae* and the nuclease-treated stabilized fermentation product of *C. somerae* were added to the diet, respectively.

**TABLE 1:** Proximate composition analysis of the fermentation product of *C. somerae* and nuclease-treated fermentation product of *C. somerae* (dry matter, g/kg)*^a^*.

Ingredient	Moisture (%)	Crude ash (%)	Crude fat (%)	Crude protein (%)	Crude fiber (%)
*C. somerae*	8.34 ± 0.21	8.60 ± 0.18	15.28 ± 0.05	15.67 ± 0.18	6.73 ± 0.03
Nuclease-treated of *C. somerae*	8.17 ± 0.06	8.97 ± 0.27	15.21 ± 0.29	13.78 ± 0.25	6.70 ± 0.08

*^a^Values are means ± SEMs, n = 3 replicates.*

### Zebrafish Husbandry and Experimental Diets

In Experiment 1, zebrafish with 0.06 ± 0.01 g average weight were randomly allocated into 6 groups. In each group, there were 3 replicate tanks and 15 fish in each tank. Zebrafish in the control group were fed with a high-fat diet and the XMX-1 groups were fed with a high-fat diet supplemented with a 5 or 10 g XMX-1/kg diet. In the XMX-1 (N) groups, zebrafish were fed with a high-fat diet supplemented with a 5 or 10 g XMX-1 (N)/kg diet. The basal dietary formulation and proximate composition analysis were given in Table 2.

**TABLE 2:** Feed formulation and proximate composition analysis of diets for zebrafish (dry matter, g/kg).

Ingredient	g/kg DM					
	
	LFD	HFD	XMX-1 5	XMX-1 10	XMX-1 (N) 5	XMX-1 (N) 10
Flour	250	200	200	200	200	200
Soybean meal	180	196	196	196	196	196
Fish meal	450	450	450	450	450	450
Choline chloride (50%)	2	2	2	2	2	2
Ca(H_2_PO_4_)_2_	20	20	20	20	20	20
Soybean oil	12	100	100	100	100	100
VC phosphate	1	1	1	1	1	1
Bentonite	61	7	7	7	7	7
Rice husk powder	10	10	5	0	5	0
30% Rice bran + 30% Soybean meal + 40%DDGS	10	10	10	10	10	10
XMX-1	–	–	5	10	–	–
XMX-1 + N	–	–	–	–	5	10
Vitamin premix*^a^*	2	2	2	2	2	2
Mineral premix*^b^*	2	2	2	2	2	2
Total	1,000	1,000	1,000	1,000	1,000	1,000
Crude protein (%)	49.44	48.46	48.14	48.12	48.02	48.45
Crude fat (%)	6.97	15.83	17.14	17.78	20.90	14.78
Crude ash (%)	13.81	13.83	14.52	14.46	14.47	14.40
Moisture (%)	3.31	2.99	3.45	3.37	2.86	2.92

*^a^Containing the following (g/kg vitamin premix): thiamine, 0.438; riboflavin, 0.632; pyridoxine.HCl, 0.908; d-pantothenic acid, 1.724; nicotinic acid, 4.583; biotin, 0.211; folic acid, 0.549; vitamin B-12, 0.001; inositol, 21.053; menadione sodium bisulfite, 0.889; retinyl acetate, 0.677; cholecalciferol, 0.116; dl-α-tocopherol-acetate, 12.632.*

*^b^Containing the following (g/kg mineral premix): CoCl_2_.6H_2_O, 0.074; CuSO_4_.5H_2_O, 2.5; FeSO_4_.7H_2_O, 73.2; NaCl, 40.0; MgSO_4_.7H_2_O, 284.0; MnSO_4_.H_2_O, 6.50; KI, 0.68; Na_2_SeO_3_, 0.10; ZnSO_4_.7H_2_O, 131.93; Cellulose, 501.09.*

In Experiment 2, 360 zebrafish (average initial mass 0.026 ± 0.0 g) were randomly divided into 12 tanks, each having three randomly assigned replicate tanks per treatment and 15 fish in each tank. In the XMX-1 and XMX-1 (N) groups, zebrafish were fed with a high-fat diet supplemented with a 100 g XMX-1/kg diet and 100 g XMX-1 (N)/kg diet, respectively. The basal dietary formulation and proximate composition analysis were given in Table 3.

**TABLE 3:** Feed formulation and proximate composition analysis of diets for zebrafish (dry matter, g/kg).

Ingredient	g/kg DM			
	
	LFD	HFD	XMX-1100	XMX-1 (N) 100
Flour	177	127	127	127
Soybean meal	180	196	196	196
Fish meal	450	450	450	450
Choline chloride (50%)	2	2	2	2
Ca(H_2_PO_4_)_2_	20	20	20	20
Soybean oil	12	100	100	100
VC phosphate	1	2	2	2
Rice husk powder	154	100	0	0
XMX-1	–	–	100	–
XMX-1 (N)	–	–	–	100
Vitamin premix*^a^*	2	2	2	2
Mineral premix*^b^*	2	2	2	2
Total	1,000	1,000	1,000	1,000
Crude protein (%)	44.32	44.98	44.71	44.53
Crude fat (%)	7.95	18.70	18.83	18.36
Moisture (%)	2.41	2.47	2.87	2.27

*^a^Containing the following (g/kg vitamin premix): thiamine, 0.438; riboflavin, 0.632; pyridoxine.HCl, 0.908; d-pantothenic acid, 1.724; nicotinic acid, 4.583; biotin, 0.211; folic acid, 0.549; vitamin B-12, 0.001; inositol, 21.053; menadione sodium bisulfite, 0.889; retinyl acetate, 0.677; cholecalciferol, 0.116; dl-α-tocopherol-acetate, 12.632.*

*^b^Containing the following (g/kg mineral premix): CoCl_2_.6H_2_O, 0.074; CuSO_4_.5H_2_O, 2.5; FeSO_4_.7H_2_O, 73.2; NaCl, 40.0; MgSO_4_.7H_2_O, 284.0; MnSO_4_.H_2_O, 6.50; KI, 0.68; Na_2_SeO_3_, 0.10; ZnSO_4_.7H_2_O, 131.93; Cellulose, 501.09.*

In both experiments, zebrafish were fed with diets at a ratio of 6% of body weight at 09:00 and 16:00 every day in a recirculating system with water temperature 28–28.5°C, pH 7.0–7.5, dissolved oxygen ≥ 6 mg/L and a 12 h/12 h light/dark cycle.

### Growth Measurements

Zebrafish were weighed at the start of the experiment. After a 3-week feeding, the fish was weighed after 24 h fasting. The survival rate, weight gain, and feed conversion rate of fish were evaluated as previously described (18).

### Determination of Total Antioxidant Capacity (T-AOC), Lysozyme, and Complement Component 4 in Skin Mucus

Skin mucus was collected from 24 fish in each group with 6 replicates. The T-AOC content was determined according to the manufacturer’s instructions using a kit (Cominbio, Suzhou, China). Assay kits were used to determine the levels of lysozyme and C4 (Jiancheng Bioengineering Ins., China).

### Determination of Liver Triacylglycerol Content

The liver samples of zebrafish were weighed and homogenized in phosphate buffered saline (PBS). The TAG was extracted according to the protocol described previously (30). To quantify TAG, free glucose reagent (Sigma–Aldrich, Shanghai) and triglyceride reagent (Sigma–Aldrich, Shanghai) were utilized. The concentrations of TAG are shown as μg/mg liver.

### Total RNA Extraction, Reverse Transcription, and *q*RT-PCR Analysis

Trizol (Invitrogen, Carlsbad, CA, United States) was used to extract total RNA from zebrafish liver and intestine. The cDNA synthesis and the quantitative real-time PCR response were conducted using the previously published procedures (30). Table 4 contains a list of primer sequences. The 2^–ΔΔ^^CT^method was used to analyze the data. The reference gene was ribosomal protein S11 (*RPS11*). GraphPad Prism 8.0 software was used to analyze the data.

**TABLE 4:** Primer sequences for qPCR.

Gene	Forward primer (5′–3’)	Reverse primer (5′–3′)
*RPS11*	ACAGAAATGCCCCTTCACTG	GCCTCTTCTCAAAACGGTTG
*TNF*-α	AAGGAGAGTTGCCTTTACCG	ATTGCCCTGGGTCTTATGG
*IL-6*	TCAACTTCTCCAGCGTGATG	TCTTTCCCTCTTTTCCTCCTG
*IL-1*β	GGCTGTGTGTTTGGGAATCT	TGATAAACCAACCGGGACA
*IL-10*	TCACGTCATGAACGAGATCC	CCTCTTGCATTTCACCATATCC
*TGF-*β	TGCGGCACCCAATCACACAAC	GTTAGCATAGTAACCCGTTGGC
*FAS*	GGAGCAGGCTGCCTCTGTGC	TTGCGGCCTGTCCCACTCCT
*ACC1*	GCGTGGCCGAACAATGGCAG	GCAGGTCCAGCTTCCCTGCG
*PPAR*α	CTGCGGGACATCTCTCAGTC	ACCGTAAACACCTGACGACG
*UCP2*	TGCCACCGTGAAGTTTATTG	CCTCGATATTTCACCGGACC
*CPT1*	GCATTGACCTTCAGCTCAGC	CTGCCAACACCAGCACGAAC
*PGC1a*	CCCCTTTGCCCTGACCTGCCTGAG	GAAGGACAGCTCTGATCACTGGCATTGG
*PPAR*γ	CCTGTCCGGGAAGACCAGCG	GTGCTCGTGGAGCGGCATGT
DGAT2	CCATACTTGCTGCATATTCC	ATGTCATGATAAACTGCAGC

### The 16S *r*RNA Gene Sequencing

Six hours following the last feeding, the gut content was taken from each treatment group. The gut microbiota was studied using 16S *r*RNA gene sequencing. The protocols for DNA extraction, sequencing, and data analysis were carried out as described previously (30). Microbiota sequencing data in this study are available from the National Center for Biotechnology Information (NCBI) under accession numbers: PRJNA 825074 (Experiment 1) and PRJNA 824236 (Experiment 2).

### Statistical Analysis

The data were presented as mean ± SEMs. GraphPad Prism 8.0 and Microsoft Office Excel 2010 software were used to generate all statistics from at least three different tests. The Student’s *t*-test was used to compare the differences between the two groups. The difference with *p*-value less than 0.05, was considered significant.

## Results

### Effects of XMX-1 and XMX-1 (N) Diets on Growth of Zebrafish

**FIGURE 1:**
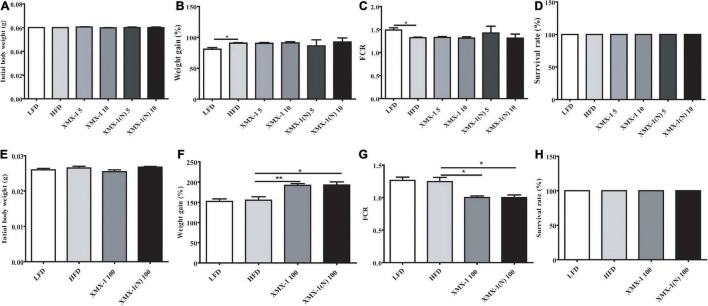
Effects of XMX-1 and XMX-1 (N) diets on zebrafish survival, weight gain (%), and feed conversion ratio. **(A)** Initial body weight (g), **(B)** weight gain (percentage), **(C)** feed conversion ratio (FCR), and **(D)** survival rate with low dosage addition. **(E)** Initial body weight (g), **(F)** weight gain (percent), **(G)** feed conversion ratio (FCR), and **(H)** survival rate with high dosage addition. The means (SEMs) (*n* = 3) were used to represent the data. *p < 0.05 and **p < 0.01 comparison to the control group.

Feeding zebrafish which have similar initial body weight (Figure 1A), a high-fat diet led to a higher weight gain and lower FCR compared with the control group (Figures 1B,C, *p* < 0.05). The addition of 5 or 10 g/kg XMX-1 and 5 g/kg or 10 g/kg XMX-1 (N) had no effect on zebrafish growth (Figures 1B,C, *p* > 0.05). Interestingly, 100 g/kg XMX-1 or 100 g/kg XMX-1 (N) supplementation significantly increased weight gain and decreased FCR of zebrafish with similar initial body weight (Figure 1E) compared with the HFD group (Figures 1F,G, *p* < 0.05). The survival rate of fish in all treatments has no significant differences compared to the HFD group (Figures 1D,H, *p* > 0.05).

### Effects of XMX-1 and XMX-1 (N) Diets on the Non-specific Immunity of Zebrafish

**FIGURE 2:**
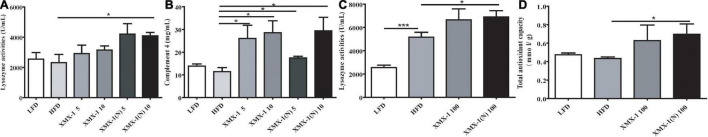
Effects of XMX-1 and XMX-1 (N) diets on zebrafish body surface mucus immunity. **(A)** Lysozyme activity and **(B)** complement component 4 with low dosage addition. **(C)** Lysozyme activity and **(D)** total antioxidant capacity with high dosage addition. The means (SEMs) (*n* = 6) were used to represent the data. *p < 0.05 and ***p < 0.001 comparisons to the control group.

Dietary 5 or 10 g/kg XMX-1, and 5 g/kg XMX-1 (N) had no effect on skin mucus lysozyme activity compared with the HFD group (Figure 2A, *p* > 0.05). Lysozyme activity increased significantly in the 10 g/kg XMX-1 (N) group as compared to the HFD group (Figure 2A, *p* < 0.05). Furthermore, the supplementation with 5 g/kg or 10 g/kg XMX-1 and 5 g/kg or 10 g/kg XMX-1 (N) significantly enhanced the level of C4 in zebrafish skin mucus (Figure 2B, *p* < 0.05). The fish fed with a 100 g/kg XMX-1 diet had no effect on lysozyme activity and the total antioxidant capacity (Figures 2C,D, *p* > 0.05). In contrast, the dietary XMX-1 (N) at 100 g/kg significantly increased lysozyme activity and total antioxidant capacity compared with the HFD group (Figures 2C,D, *p* < 0.05).

**FIGURE 3:**
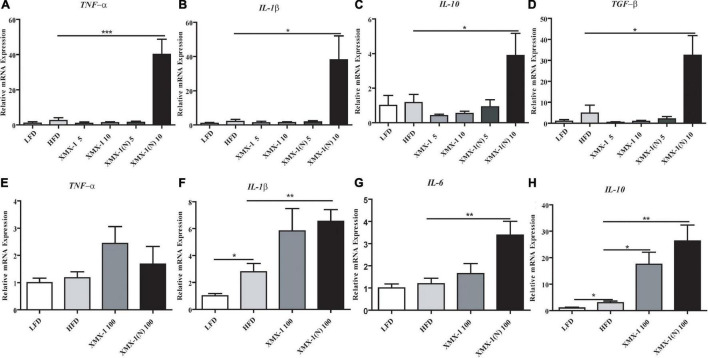
Effects of XMX-1 and XMX-1 (N) diets on the expression of intestinal inflammation-related genes of zebrafish. **(A)** *TNF-*α, **(B)** *IL-1*β, **(C)** *IL-10*, and **(D)** *TGF-*β with low dosage addition. **(E)** *TNF-*α, **(F)** *IL-1*β, **(G)** *IL-6*, and **(H)** *IL-10* with high dosage addition. The means (SEMs) (*n* = 6) were used to represent the data. *p < 0.05, **p < 0.01, and ***p < 0.001 comparison to the control group.

The expression of inflammation-related genes such as tumor necrosis factor alpha (*TNF-*α), interleukin 1 beta (*IL-1*β), interleukin 10 (*IL-10*), and transforming growth factor beta (*TGF-*β) was significantly higher in the 10 g/kg XMX-1 (N) group (Figures 3A–D, *p* < 0.05). Moreover, feeding 100 g/kg XMX-1, didn’t affect *TNF*-α expression (Figure 3E, p > 0.05) but significantly increased the expression of *IL-10* (Figure 3H, *p* < 0.05), while the expression of *IL-1*β, *IL-10*, and interleukin 6 (*IL-6*) was significantly increased in the 100 g/kg XMX-1 (N) group compared with the HFD group (Figures 3F–H, *p* < 0.01).

### Effects of XMX-1 and XMX-1 (N) Diets on the Gut Microbiota of Zebrafish

**FIGURE 4:**
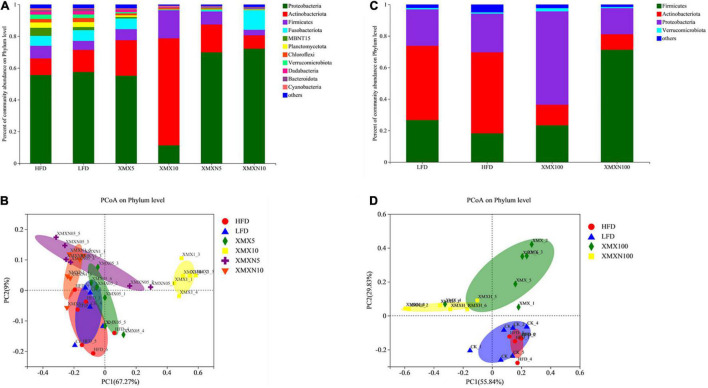
Effects of XMX-1 and XMX-1 (N) diets on the gut microbiota of zebrafish. **(A)** Relative abundance; **(B)** principal coordinates analysis (PCoA) at phylum level of the gut microbiota with low dosage addition. **(C)** Relative abundance; **(D)** PCoA at phylum level of the gut microbiota with high dosage addition.

Figure 4A shows that supplementation with 5 and 10 g/kg XMX-1 increased the relative abundance of Actinobacteria compared with the HFD group. Dietary XMX-1 at 10 g/kg reduced the relative abundance of Proteobacteria while increased the relative abundance of Firmicutes. Proteobacteria, Actinobacteria, and Firmicutes were found to be more abundant in the 5 g/kg XMX-1 (N) treatment. A 10 g/kg XMX-1 (N) increased the relative abundance of Proteobacteria and Fusobacteria. Furthermore, there were significant differences among groups based on PCoA analysis, indicating that the XMX-1 and XMX-1 (N) diets had substantial effects on the autochthonous microbiota (Figure 4B, *p* < 0.05).

Dietary 100 g/kg XMX-1 enhanced the relative abundance of Firmicutes and Proteobacteria while decreased the relative abundance of Actinobacteria (Figure 4C). The supplementation 100 g/kg XMX-1 (N) enhanced the relative abundance of Firmicutes while decreased the relative abundance of Proteobacteria and Actinobacteria (Figure 4C). Furthermore, PCoA analysis revealed substantial differences among groups, indicating that 100 g/kg XMX-1 and 100 g/kg XMX-1 (N) diets had significant effects on the autochthonous microbiota compared with HFD control (Figure 4D, *p* < 0.05).

### Effects of XMX-1 and XMX-1 (N) Diets on the Liver Health of Zebrafish

**FIGURE 5:**
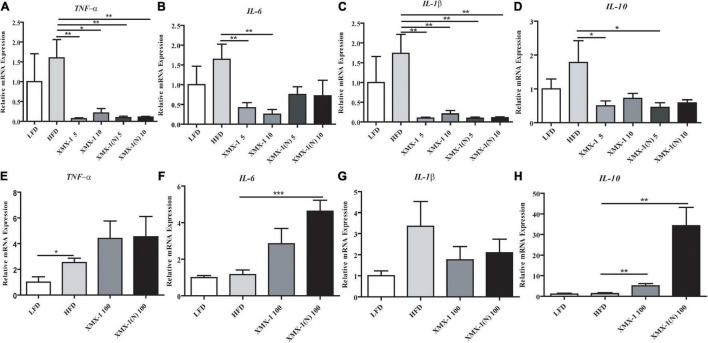
Effects of XMX-1 and XMX-1 (N) diets on the expression of liver inflammation-related genes of zebrafish. **(A)** *TNF-*α, **(B)** *IL-6*, **(C)** *IL-1*β, and **(D)** *IL-10* with low dosage addition. **(E)** *TNF-*α, **(F)** *IL-6*, **(G)** *IL-1*β, and **(H)** *IL-10* with high dosage addition. The means (SEMs) (*n* = 6) were used to represent the data. *p < 0.05, **p < 0.01, and ***p < 0.001 comparison to the control group.

As can be seen in Figure 5, dietary 5 or 10 g/kg XMX-1 and 5 or 10 g/kg XMX-1 (N) reduced the expression of inflammation-related genes in zebrafish liver compared with the HFD group (Figures 5A–D, *p* < 0.05). In contrast, the fish fed with a 100 g/kg XMX-1 diet increased *IL-10* expression (Figure 5H, *p* < 0.01). Furthermore, *IL-6* and *IL-10* expressions were considerably elevated in the 100 g/kg XMX-1 (N) diet group (Figures 5F,H, *p* < 0.01).

**FIGURE 6:**
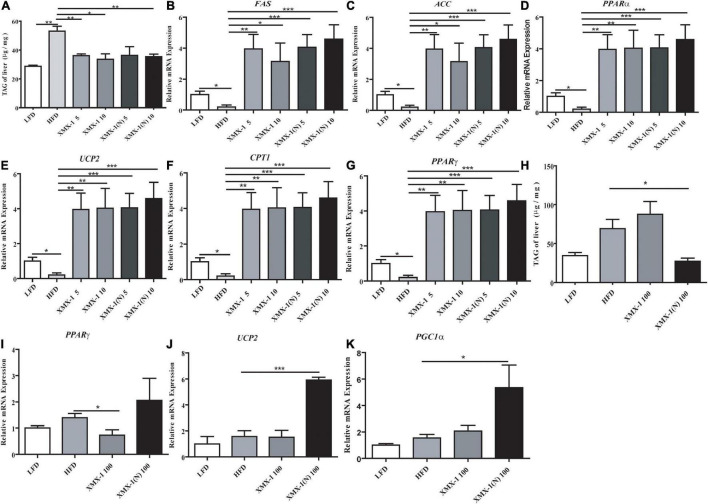
Effects of XMX-1 and XMX-1 (N) diets on the expression of the content of TAG and hepatic lipid metabolism related genes of zebrafish liver. **(A)** TAG, **(B)** *FAS*, **(C)** *ACC*, **(D)** *PPAR*α, **(E)** *UCP2*, **(F)** *CPT1*, and **(G)** *PPAR*γ with low dosage addition. **(H)** TAG, **(I)** *PPAR*γ, **(J)** *UCP2*, and **(K)** *PGC1*α with high dosage addition. The means (SEMs) (*n* = 6) were used to represent the data. *p < 0.05, **p < 0.01, and ***p < 0.001 comparison to the control group.

The TAG content of zebrafish liver was significantly reduced in the 5 or 10 g/kg XMX-1 groups and the 5 or 10 g/kg XMX-1 (N) groups compared with the HFD group (Figure 6A, *p* < 0.05). In addition, we detected the expression of genes involved in lipid metabolism. In comparison to the HFD group, the addition of 5 or 10 g/kg XMX-1 and 5 or 10 g/kg XMX-1 (N) significantly increased the expression of genes such as fatty acid synthase (*FAS*), acetyl-CoA carboxylase (*ACC*), peroxisome proliferator-activated receptor gamma (*PPAR*γ), uncoupling protein 2 (*UCP2*), carnitine palmitoyl transferase 1 (*CPT1*), and peroxisome proliferator-activated receptor alpha (*PPAR*α) (Figures 6B–G, *p* < 0.05).

Compared with the HFD group, 100 g/kg XMX-1 (N) significantly decreased the content of TAG of zebrafish (Figure 6H, *p* < 0.05). Moreover, the dietary 100 g/kg XMX-1(N) didn’t affect *PPAR*γ expression (Figure 6I, *p* > 0.05) but significantly increased the expression of lipolysis genes such as *UCP2* and proliferator-activated receptor γ coactivator 1α (*PGC1*α) (Figures 6J,K, *p* < 0.05).

## Discussion

In the present work, feeding *C. somerae* XMX-1 improved the non-specific immunity and liver health, as well as reduced lipid deposition in HFD-diet zebrafish. In addition, the effect of XMX-1 supplementation on fish growth was dose dependent. These favorable effects of XMX-1, along with the previous results (18, 19) indicate that the fermentation product of *C. somerae* has a great potential for improving fish health when used as feed additives. To further improve the function of the fermentation product of *C. somerae*, nuclease was used to treat the fermentation product and produce nucleotides. Compared to XMX-1, the concentration of nucleotides was elevated by 31.67% after the treatment of nuclease in XMX-1 (N). It has been reported that the dietary nucleotides can enhance growth, increase stress and disease resistance, modulate immune functions, and improve intestinal morphology and gut microbiota of fish (28). In particular, nucleotides have been reported to enhance growth in zebrafish (29), grouper (*Epinephelus malabaricus*) (31), red drum (*Sciaenops ocellatus*) (32), amberjack (*Seriola dumerili*) (33) and Nile tilapia (*Oreochromis niloticus*) (34). Interestingly, dietary 100 g/kg XMX-1 (N) significantly increased weight gain and decreased FCR compared to the HFD diet, while supplementation with low levels of *C. somerae* XMX-1 (N) at 5 or 10 g/kg had no influence on the growth of zebrafish. The dose effect of XMX-1 (N) on growth performance is correlated with the dose response of dietary nucleotides on growth of fish. For instance, Tahmasebi-Kohyani et al. added different levels of supplemented nucleotides (0.5, 1, 1.5, and 2 g/kg) to the diet of rainbow trout (*Oncorhynchus mykiss*) and found that the percentage of body weight gain and feed efficiency of fish was better when the fish were fed with 1.5–2 g/kg diets (35). Notably, the nucleotide levels in 5–100 g/kg XMX-1 (N) are in a similar range as the doses used in the studies investigating dietary nucleotides (33–35). Therefore, the nucleotides may contribute to the beneficial effects of nuclease-treated *C. somerae*. There are some explanations for how nucleotides work to improve fish growth, such as being feed attractants (36), supporting high rate of cell replication of fish (28), higher hepatic insulin-like growth factor 1 (*IGF-1*) gene expression (37), and reduced standard metabolic rate (38).

Fish live in an aqueous environment that is rich in pathogens, and lysozyme is critical in the defense against infection (39–41). Complement activity can trigger cellular defense and affect fish phagocytosis (42). In the current study, higher lysozyme activity and C4 were observed in the fish fed with an XMX-1 (N) diet than in fish on the HFD diet, and dietary 100 g/kg XMX-1 (N) significantly enhanced the antioxidant capacity of zebrafish. Dietary nucleotides have been reported to increase the lysozyme and complement the activities in common carp (43). Cheng et al. found that red drum fed with 5 g/kg of nucleotides had higher lysozyme activity (44). In addition, nucleotides supplementation can improve the oxidative stress resistance of fish (33, 37). Xu et al. showed that juvenile hybrid tilapia (*Oreochromis niloticus*♀ × *O. aureus*♂) fed with 6 g/kg nucleotides had higher superoxide dismutase activity and lower malondialdehyde levels than the control fish, indicating an improvement in the antioxidant status (45). Moreover, the current study also demonstrated that the expression of inflammation-related genes was significantly higher in the XMX-1 (N) diet group compared to the HFD group. In line with our results, Reda et al. discovered that the addition of nucleotides (0.5, 1.5, and 2.5 g/kg) increased the intestinal cytokine *TNF-*α, *IL-1*β, *IL-10*, and *TGF-*β and had a dosage effect compared to the control (34). Notably, the dose effect of XMX-1 (N) on lysozyme activity, antioxidant capacity, and cytokines expression is consistent with the dose responses of dietary nucleotides reported in the previous studies (44–46). These findings suggest that the dietary supplementation with XMX-1 (N) can promote a non-specific immunity in zebrafish and the nucleotides generated by the nuclease treatment of *C. somerae* fermentation product may play a role in this process.

Gut microbiota plays a key role in regulating gene expression of the digestive tract, promoting nutrition metabolism, and innate immune response (47). In the present work, PCoA analysis revealed substantial differences across groups, indicating that XMX-1 (N) diets had significant effects on the autochthonous microbiota. Specifically, 100 g/kg XMX-1 (N) supplementation enhanced the relative abundance of Firmicutes while decreased the relative abundance of Proteobacteria and Actinobacteria. Guo et al. reported that the nucleotide-fed zebrafish exhibited a higher abundance of Fusobacteria and a lower abundance of Proteobacteria than the control group (38). Furthermore, *Pedicoccus acidilactici* has been reported to successfully colonize the digestive tract of goldfish (*Carassius auratus*) fed with 5 g/kg nucleotides supplemented diets (48). These findings suggest that the nucleotides may contribute to the gut microbiota modulation effect of XMX-1 (N), which awaits further investigation.

Notably, the three doses of XMX-1 (N) supplementation all lowered the level of TAG in zebrafish liver. The previous studies have shown that the dietary nucleotides improved hepatic function and reduced hepatic lipid when compared to a control group, indicating that the dietary nucleotides influenced hepatic lipid deposition (49–52). Consistent with these results in mammals, the improvement in body lipid contents and hepatosomatic index can be observed in red sea bream (*Pagrus major*) fed with nucleotides diets (37). Interestingly, Ran et al. found that 1 g/kg nucleotides supplementation lowered the level of TAG in the liver and decreased the expression of genes involved in lipid synthesis while increased the expression of genes involved in fatty acid oxidation in zebrafish (29). In the present work, the nucleotides levels in the three XMX-1 (N) groups (0.29, 0.58, 5.8 g/kg, respectively) are in a similar range as previous studies (29, 38), suggesting the relative contribution of nucleotides to the hepatic lipid-lowering effect.

## Conclusion

The current findings suggest that the dietary XMX-1 (N) at 100 g/kg improved zebrafish growth. Three doses of XMX-1 (N) supplementation enhanced non-specific immunity. In addition, 100 g/kg XMX-1 (N) supplementation improved zebrafish gut microbiota and reduced hepatic lipid deposition. These findings indicate that the nuclease-treated *C. somerae* XMX-1 fermentation product improves fish growth and health, implying that it might be used as a functional feed additive in fish culture.

## Data Availability Statement

The datasets presented in this study can be found in online repositories. The names of the repository/repositories and accession number(s) can be found below: https://www.ncbi.nlm.nih.gov/, PRJNA 825074; https://www.ncbi.nlm.nih.gov/, PRJNA 824236.

## Ethics Statement

The animal study was reviewed and approved by the Institute of Feed Research of Chinese Academy of Agricultural Sciences, which was chaired by the China Council for Animal Care (Assurance No. 2018-AF-FRI-CAAS-001).

## Author Contributions

MX and QH: formal analysis, data curation, writing original draft preparation, and revising the draft. RX: methodology and investigation. RO: resources and methodology. ER: analysis and methodology. YY and ZZ: analysis and resources. CR: data curation, project administration, and funding acquisition. ZGZ: project administration, supervision, and funding acquisition. All authors contributed to the article and approved the submitted version.

## Conflict of Interest

The authors declare that the research was conducted in the absence of any commercial or financial relationships that could be construed as a potential conflict of interest.

## Publisher’s Note

All claims expressed in this article are solely those of the authors and do not necessarily represent those of their affiliated organizations, or those of the publisher, the editors and the reviewers. Any product that may be evaluated in this article, or claim that may be made by its manufacturer, is not guaranteed or endorsed by the publisher.
